# Cytomegalovirus Biology Viewed Through a Cell Death Suppression Lens

**DOI:** 10.3390/v16121820

**Published:** 2024-11-23

**Authors:** Edward S. Mocarski

**Affiliations:** 1 Department of Microbiology & Immunology, Stanford Medical School, Stanford University, Stanford, CA 94305, USA; mocarski@stanford.edu; 2 Department of Microbiology & Immunology, Emory Medical School, Emory Vaccine Center, Emory University, Atlanta, GA 30322, USA

**Keywords:** herpesvirus, apoptosis, necroptosis, pyroptosis, viral suppression

## Abstract

Cytomegaloviruses, species-specific members of the betaherpesviruses, encode an impressive array of immune evasion strategies committed to the manipulation of the host immune system enabling these viruses to remain for life in a stand-off with host innate and adaptive immune mechanisms. Even though they are species-restricted, cytomegaloviruses are distributed across a wide range of different mammalian species in which they cause systemic infection involving many different cell types. Regulated, or programmed cell death has a recognized potential to eliminate infected cells prior to completion of viral replication and release of progeny. Cell death also naturally terminates replication during the final stages of replication. Over the past two decades, the host defense potential of known programmed cell death pathways (apoptosis, necroptosis, and pyroptosis), as well as a novel mitochondrial serine protease pathway have been defined through studies of cytomegalovirus-encoded cell death suppressors. Such virus-encoded inhibitors prevent virus-induced, cytokine-induced, and stress-induced death of infected cells while also moderating inflammation. By evading cell death and consequent inflammation as well as innate and adaptive immune clearance, cytomegaloviruses represent successful pathogens that become a critical disease threat when the host immune system is compromised. This review will discuss cell death programs acquired for mammalian host defense against cytomegaloviruses and enumerate the range of modulatory strategies this type of virus employs to balance host defense in favor of lifelong persistence.

## 1. Overview

Cytomegaloviruses (CMVs) are characterized by their highly restricted host range, widespread distribution and coevolution with mammalian host species [1,2,3,4,5,6]. These members of the betaherpesvirus subgroup of herpesviruses retain a common, species-restricted biology with little disease observed in immunocompetent individuals and risk of opportunistic disease in immunocompromised hosts as well as congenital disease when passed across the placenta during pregnancy [7,8]. CMV gene products orchestrate this lifestyle, including lifelong systemic infection and ubiquitous distribution by controlling critical signal transduction pathways in infected cells and impacting the way that the antiviral immune response unfolds. Study of the human CMV (HCMV) balance with its human host has unveiled potential insights into disease pathogenesis, for example, into congenital disease as well as potential for serious illness in immunocompromised individuals [8,9,10]. When first identified, sequence homologs of host immune proteins encoded by HCMV have long been suspected of orchestrating pathogenesis [11,12]. Following systematic direct mutagenesis [13,14], a majority of annotated viral proteins were found dispensable for viral replication. These “nonessential” gene products form the foundation of an ever-expanding catalog of virus-encoded immune modulators that deflect a variety of immune functions while surprisingly enhancing others, all to presumably advance the opportunistic interests of the virus [8,9,10,15,16,17,18]. Elaboration of the function of these individual gene products presents a major success story even though the relative importance of each immune modulator and its immune target largely remains a mystery due to limitations imposed by species specificity. Innate and adaptive immune mechanisms are conserved across mammals, so CMVs infecting various laboratory mammals reveal the diversity of immune evasion and exploitation strategies that manipulate host responses [9,15,19,20,21,22,23]. Exactly why the evolution of such virus–host partnerships favored the adaptation of such diverse and novel evasion strategies to counteract conserved host defense mechanisms remains an outstanding question even though the remarkable success of CMVs within each of their respective hosts is a testament to the value of such concerted immunomodulation to this pathogen [10,21].

The large commitment CMV makes to subverting host defense is evident in its genome. Only one-third of the 180 annotated protein- and long RNA-encoding HCMV genes contribute to viral replication in cell culture [8,10,13,14]. Adding to this list virus-encoded microRNAs [24,25,26], detailed transcriptional mapping [27] and systematic profiling of genome protein-coding potential [28,29] together demonstrate that the HCMV functional repertoire is huge as well as diverse. The enormous number of HCMV genes that are dispensable for replication [8,10,12,16,17,18] plus the fact that these act to modulate all levels of host defense [9,15,17,18,21,30,31] begs for experimental approaches to decipher their functional hierarchy as determinants of pathogenesis in the natural human host. While investigating the activities of individual HCMV-encoded genes has yielded clues, understanding the overall impact of immune modulators in humans remains challenging. Human studies [32,33] performed with spontaneous deletion mutants [34] or with chimeric viruses carrying different sets of mutations [35] certainly revealed patterns of attenuation long before the function of the individual mutated genes was explored. Human clinical trials of live attenuated HCMV vaccine candidates [36,37,38,39] certainly, reinforce the delicate balance determined by the host immune system and pathogen escape and exploitation strategies while also alerting to the presence of mixtures of viral strain variants in some clinical studies [40,41]. In all, this research has sketched a signature of a highly successful and intensively manipulative human pathogen [42,43,44] that exhibits an unstable genome upon propagation in cultured cells [45,46,47,48]. Together with HCMV, CMVs infecting mice, murine cytomegalovirus (MCMV), guinea pigs (GPCMV), and monkeys, including rhesus macaque cytomegalovirus (RhCMV), have provided insights into virus biology. All exhibit similarity but, curiously, even close great ape relatives of HCMV often employ distinct modulatory mechanisms to counterbalance conserved host defense pathways [21,22,49,50,51,52]. Studies with MCMV, in particular, have elucidated principles of infection, tissue tropism, immune response, and immune modulation facilitated by a tractable mammal where mutagenesis of immune pathways is technically feasible in both the virus and the host [9,15,17,19,20,21,53]. There remains little doubt that, during mammalian evolution, CMVs retained common biological properties through the acquisition of diverse modulatory mechanisms targeting distinct points in host defense signaling to accomplish the pathogen-host balance that exists today.

HCMV is distributed widely across the world’s populations. Only humans are naturally susceptible. Great ape CMVs have coevolved with their respected host lineages [3] and show dramatic diversity as well as species specificity such that even chimpanzee cytomegalovirus (CCMV) represents a recognizable and distinct virus lineage [6,54]. RhCMV, an important surrogate model of HCMV infection and antiviral immunity, has been employed for experimental infection in macaques [49,50,52,55]. When certain immune evasion genes are disrupted, RhCMV gains the ability to infect a different old-world monkey species that are not susceptible to the non-mutated parental virus [56]. While this nonhuman primate CMV is not known to infect humans, human cells are permissive and support full replication [57]. Overall, the coevolutionary process involving CMVs shows fewer species jumps than observed with other herpesviruses [3,6]. With most nonhuman CMVs, such as MCMV, infection of the natural host is universal in the wild [58]. While closely related species such as rats appear susceptible to experimental MCMV infection [7], infection of human cells results in entry and early gene expression but is nonproductive, evident as a post-entry species restriction [57,59]. The inability of MCMV to replicate in human cells is overcome by suppressing programmed cell death pathways through transduction of either human BCL2-like cell death suppressors such as human BCL_XL_ or HCMV-encoded UL37x1 into cells [60]. Mutations in a different MCMV genetic locus, M112-113, encoding a DNA replication function (E1) also increase the susceptibility of human cells [61]. Surprisingly, the species restriction is associated with markers of mitochondrial apoptosis with MCMV M112-113 mutants, revealing possibilities that “MCMV mitochondrial apoptosis inhibitors have insufficient activity in human cells” or “MCMV exerts a stronger proapoptotic stress on human cells than on mouse cells” to dictate restriction [62]. This type of study begins to unveil species restriction rules for CMVs but has not yet been extended to HCMV or any other CMVs. Nevertheless, existing evidence provides a good example of the intricate and likely ongoing coevolutionary relationship between CMVs and their various natural mammalian hosts [1,54].

## 2. Programmed Cell Death Pathways in Antiviral Host Defense

Programmed (or regulated) cell death signaling is recognized as an ancient form of host defense [63,64]. Over their evolution, herpesviruses acquired means to delay or block cell death signaling to complete or prolong replication and disseminate while controlling inflammation [9,19,65,66,67,68]. CMVs deploy cell death suppressors to counterbalance host immune clearance mechanisms. Betaherpesviruses have enjoyed tens of millions of years of coevolution as their hosts adapted ever-more elaborate antiviral immune mechanisms by successively deploying inhibitors of mitochondrial death pathways, caspase-8-mediated apoptosis, necroptosis, and pyroptosis [19,68,69,70]. CMVs, together with other herpesviruses and poxviruses, contribute to host deployment of ever more elaborate cell death pathways over the course of chordate evolution [63,68,71,72]. Through maintaining a balance, herpesviruses achieve dissemination in populations as well as individuals, infecting and persisting in varied cell types without being completely eliminated during the life of the host [3,6,62,73]. While viral immune modulators are typically most effective in their native host species, over the 80 million years primates and rodents have diverged from a common ancestor, HCMV and MCMV and their betaherpesvirus relatives retain homologs capable of suppressing mitochondrial as well as caspase-8-mediated apoptosis across species lines [69].

Programmed cell death was initially recognized for the role apoptosis plays in eliminating excess cells in multicellular organisms [74,75,76]. Given the existence of intrinsic apoptosis machinery in yeast and other single-cell eukaryotes, this form of death predates the emergence of multicellular organisms and manifests as a form of host defense in single-celled organisms [63,77,78,79]. The recognition that killer RNA in yeast induces apoptosis and that viruses infecting multicellular organisms encode a wide variety of inhibitors known to block apoptosis supports this host defense role for apoptosis [80,81,82,83,84,85,86]. The same might be the case for pathogen-triggered pyroptosis [63]; however, the repurposing of cell death from host defense to developmental and homeostatic purposes remains an important incompletely resolved topic [87,88].

Apoptosis in chordates is widely recognized to initiate from various internal stress signals and eliminates cells via prescribed deployment of proapoptotic and antiapoptotic mitochondrial BCL2 family members [89]. In vertebrates, the main intrinsic apoptotic pathway depends on BAX- and BAK-mediated mitochondrial outer membrane permeabilization (MOMP) thereby releasing components such as cytochrome *c* that trigger caspase-9-mediated proteolytic cleavage-activation of executioner caspase-3, caspase-6 or caspase-7 [90]. Activation of executioner caspases drives nuclear changes and cell dismemberment through proteolytic cleavage/activation of cellular targets [88,89,90,91,92]. Fragmenting cells are recognized and engulfed by professional phagocytes such as macrophages and dendritic cells without inciting inflammation [93,94]. Studies in developmental and homeostatic settings support a noninflammatory process as the principal means of removing excess cells throughout development and life. Importantly, in uninfected tissues, the removal of apoptotic cells is accomplished prior to the leakage of proinflammatory components. In contrast to the homeostatic elimination of excess or unneeded cells, apoptosis of virus-infected cells proceeds in an environment that fosters an antiviral immune response with viral antigen presentation and inflammatory cytokine deployment, all in association with inflammation and disease outcomes [63,64,95,96,97,98].

As originally characterized, extrinsic apoptosis [89,99,100] represents a pathway initiated downstream of TNF family death domain-containing receptors, proceeding via caspase-8 autoactivation and either direct activation of executioner caspase-3, caspase-6 or caspase-7, or via an intermediary step where BCL2 family member BID, a target of caspase-8 cleavage, amplifies signaling through BAX and BAK through a cascade indistinguishable from intrinsic apoptosis that results in activation of executioner caspases. Extrinsic apoptosis is now more broadly viewed as a caspase-8 (or caspase-10)-regulated signaling pathway [64,88] often initiated independently of TNF family death receptors and mediated via receptor-interacting protein (RIP) kinase (RIPK)1 and/or RIPK3 [101,102]. These two protein kinases dictate the balance of cell death-dependent and cell death-independent signal transduction outcomes [64,88,91,92]. Unexpectedly, and still not fully appreciated, when unleashed necroptosis is controlled in mice by elimination of RIPK3, caspase-8-deficiency contributes little to mammalian development or homeostasis and makes only a modest impact on immune response parameters by restricting the expansion of lymphocytes responding to viral infection [103,104,105,106,107,108,109]. Through such seminal work focused on MCMV-infected mice, the number of pathologic regulated cell death pathways has expanded to include necroptosis as well as, more recently [70,110], pyroptosis, and other less well-defined death pathways contributing to inflammatory death [111]. Cytokines and inflammasomes serve to amplify the inflammatory environment and broaden the range of cell death pathways associated with the disease with apoptosis certainly contributing to inflammation, along with bona fide lytic death pathways, necroptosis and pyroptosis [63,70]. The extensive innate immune crosstalk that takes place between cell death pathways and inflammatory cytokine networks has the potential to eliminate virus-infected cells and limit the spread of infection.

The alternative outcomes of extrinsic apoptosis, necroptosis, or inflammasome-mediated signaling are dictated by a constellation of inhibitors deployed by pathogens as well as the cell types susceptible to infection. Both necroptosis [68] and inflammasome-mediated pyroptosis [70,112,113,114], executed through gasdermin family pore-forming proteins [115,116], drive leakage of cytosolic contents and so are considered proinflammatory pathways [70,117,118]. Importantly, virus-infected cells may experience any of these host defense pathways if not blocked by virus-encoded cell death suppressors. PANoptosis, first noted in the setting of influenza virus-infected macrophages [119,120] refers to cells showing markers of multiple pyroptosis, apoptosis, and necroptosis (PAN) pathways that often accompany virus-induced cell death along with death-independent inflammatory signaling, potentially under the control of an amalgamated death complex, a PANosome [121,122]. Whether or not such an uber-complex executes simultaneous death pathways in individual cells remains controversial and unresolved. Studies of viral infection have documented alternate, interdependent programmed cell death pathways while also influencing cytokine signal transduction independently of dying cells that play out in different cell types suggesting that alternate cell death choices play out in nature [63,68,123,124]. The remainder of this review will focus on CMV-encoded cell death suppressors, gene products that affirm the critical importance of specific cell death pathways in antiviral host defense, complementing and providing perspective on previous publications [19,62,67,68,69,70,109,123,125,126,127,128,129]. CMVs represent truly ancient immunomodulatory tricksters engaging their hosts in an elaborate partnership through the deployment of cell death signaling inhibitors.

## 3. Apoptotic Pathways Targeted by CMV-Encoded Inhibitors of Cell Death

Apoptosis has long been known to be targeted by mammalian virus-encoded cell death suppressors, acting in concert with cytokine signaling modulators to impact the timing and type of cell death as well as inflammatory signal transduction [19,83,84,85,86]. The size and universality of this armamentarium first brought to light through amino acid sequence homology with cellular homologs, provided incontrovertible evidence that the larger the DNA virus, the greater the commitment to countering and balancing cell death pathways [19]. Many additional virus-encoded cell death suppressors have been recognized through functional assays, such as a highly potent HCMV-encoded inhibitor of BCL2 family member BAX [130], named viral mitochondrial inhibitor of apoptosis (vMIA), and the viral inhibitor of caspase-8 activation (vICA) [131]. All DNA viruses are equipped with cell death suppressors, with multiple inhibitors encoded by herpesviruses and poxviruses [19,64,68,132,133]. In addition to the catalog of viral gene products that are homologous to cellular components and/or that exhibit the ability to block death induced in experimental assays, a second powerful strategy seeks to characterize the particular death pathway targeted by an individual cell death suppressor by evaluating pattern(s) of death induced by mutant viruses carrying singly disrupted viral cell death suppressors. Such an evaluation reveals novel death pathways and applied to CMVs, has resulted in a significant expansion of programmed cell death pathways over the past 25 years.

Thus, the identification of numerous proteins and long noncoding RNAs in mammalian herpesviruses and poxviruses capable of blocking cell death posed a follow-on question: What quantitative or qualitative purpose does each of these suppressors serve either during infection of susceptible cells or when infecting a host animal? Evaluation of CMVs lacking individual cell death suppressors has proceeded to reveal novel cell death pathways and to drive home the importance of cell death in innate immunity to virus infection [19,68,69,72,134]. The unique value of studies in CMVs has been the ability to study cell type-specificity of various pathways leading to virus-induced cell death and, due to the evolutionary conservation of both cell death pathways and virus-encoded cell death suppressors in humans and mice as well as HCMV and MCMV [68,72]. A sense of hierarchy of cell death pathways and the use of alternative cell death pathways in host defense is becoming understood.

## 4. Mitochondrial Localized Inhibitors of Intrinsic Apoptosis

HCMV UL37x1-encoded vMIA prevents intrinsic apoptosis by counteracting the function of proapoptotic BCL2 family member BAX to prevent mitochondrial outer membrane permeabilization (MOMP) [130]. This immediate early cell death suppressor is encoded by exon 1 of unspliced transcripts as well as larger spliced gene products with vMIA located at the amino terminus [130,135]. Even though UL37x1 protein expression starts early after infection, its impact on cell death is only evident at intermediate to late times of infection. Two recognized vMIA protein domains are responsible for suppression initiated by mitochondrial BCL2 family protein signaling. One domain directs localization of the protein to mitochondria [69,136,137,138] as well as junctions between mitochondria and endoplasmic reticulum that have been denoted the mitochondrion-associated membranes (MAMs) [139,140,141]. The second, alpha-helical domain comprising amino acids 131 to 150 of this 163 amino acid protein dictates a direct interaction with BCL2 family member BAX and is primarily responsible for vMIA suppression of apoptosis [136,142,143,144]. The interaction with BAX is distinct from cellular cell death suppressors BCL2 and BCLxL but the impact prevents BAX oligomerization and MOMP, a key step in intrinsic apoptosis [145]. HCMV relatives such as RhCMV encode significantly condensed versions of vMIA that retain these two critical domains [69,146]. HCMV vMIA is recognized for preventing BAX activation while localized to the mitochondrial surface [66,132,142,143,144,147] while increasing proteasomal degradation of BAX as well [148]. Overall, HCMV-encoded vMIA is recognized as one of the most potent suppressors of intrinsic apoptosis known [132]. Detailed characterization also revealed autophagy as a cytoprotective pathway that may sensitize to apoptosis [149,150]. By preventing MOMP, release of cytochrome *c*, and activation of caspase-9 together with executioner caspases, vMIA completely prevents initiation of mitochondrial, stress-induced apoptosis.

Localization of vMIA to mitochondria also disrupts the interconnected reticulum and drives mitochondrial fragmentation (fission) [137,151,152,153]. Elongated, interconnected mitochondria characterize normal cells owing to the concerted and constitutive function of BAX and BAK. Unlike cell death suppression, which is primarily due to interaction with BAX, vMIA alters BAK as well as BAX to promote mitochondrial fission [151]. Thus, punctate, fragmented mitochondria are observed in all HCMV-infected cells as well as cells where vMIA is expressed independently of virus infection. This fragmentation is not observed in cells infected with UL37x1 mutant viruses where mitochondria retain a reticular network characteristic of uninfected cells [137]. Importantly, such changes to mitochondrial morphology occur even though BAX and BAK are recruited by vMIA to the mitochondrial surface [151,152] without compromising mitochondrial metabolic activity and ATP production [137,138,151,152,153,154].

Comparisons of HCMV UL37x1 and UL37x1-deficient viruses have disagreed on phenotypes ascribed to vMIA alone due to reliance on different viral strains for studies. Initial single gene knock-out mutagenesis deployed to study UL37x1-encoded vMIA was performed with a HCMV strain (AD169) lacking some 19 annotated genes [1,34,155] plus an additional inactivating point mutation unveiled an initial characterization of the UL36-encoded vICA, a potent inhibitor of caspase-8-mediated (extrinsic) apoptosis [131]. This indispensable role in viral replication [13,14,156] was not affirmed when a different vICA-expressing parental and UL37x1-deficient HCMV were compared [138,154] and shown to replicate to similar overall levels influenced by infection conditions [157]. The dramatic differences in phenotype between parental viruses can be ascribed to vICA deficiency of the AD169 strain [138]. Additional investigations with the replication-deficient AD169 mutant virus showed alterations to other cellular organelles, modified ATP metabolism, and additional infected cell cytopathic effects beyond mitochondrial fragmentation [153,158,159,160,161,162,163,164,165] which should all be evaluated with replication-competent strain of HCMV. The confounding impacts of adventitious mutations carried by laboratory strains of HCMV are now widely recognized [10,166] and bacmids re-engineered to express a full complement of viral gene products seek to address such confounding issues [46,166]. Most, but not all contemporary studies that employ HCMV mutagenesis recognize the importance of using viral strains with a minimum number of additional mutations and to fully sequence all recombinant genomes, although the area continues to be problematic.

vMIA inhibits mitochondrial apoptosis through inhibition of BAX function [130,142,151] and clearly contributes to protection from stress-induced intrinsic apoptosis of virus-infected cells [138]. Although HCMV UL37x1 does not directly contribute to replication, UL37x1-deficient virus-infected cells die several days earlier due to a caspase-independent cell death pathway [69,154,157]. Because this premature death arises late in the infection cycle, the levels of viral progeny within mutant virus-infected cells are not compromised even though production and release of progeny virus only continue for days rather than over a week. This CMV-infected cell-specific programmed cell death (cmvPCD) is under the control of mitochondrial serine protease HtrA serine peptidase 2 (HtrA2), also called Omi [167]. Suppression by vMIA essentially prolongs infected cell viability as virus-producing factories [154]. Curiously, the same death pathway terminates infection of vMIA-expressing viruses suggesting a transient impact of vMIA. HtrA2/Omi has long been recognized as an accessory executioner during apoptosis [167,168]. Studies comparing UL37x1 mutant and parental vMIA-expressing viruses indicate cmvPCD proceeds without a requirement for mitochondrial release of this protease [69,154,157]. Overall, vMIA extends the life of infected cells and likely promotes fitness during infection, particularly given the behavior of mutant viruses disrupting MCMV orthologs of vMIA [134].

Identification of analogous MCMV cell death suppressors was facilitated by bioinformatic prediction of actively translated protein-coding regions within rodent CMV genomes [169]. Out of this effort, the function of two previously unrecognized MCMV open reading frames, m38.5 and m41.1, emerged [138,170,171,172]. MCMV-encoded m38.5 localizes to and induces fragmentation of mitochondria, with potent inhibition of intrinsic apoptosis by interacting with BAX in mouse cells, activities that are analogous to HCMV UL37x1 even though the two proteins share little amino acid sequence homology [138,152,154,157,170,171,173]. Thus, the MCMV m38.5-encoded protein, called vMIA [138], targets BAX function at the mitochondrial surface much like HCMV vMIA [170,171,173]. In addition, MCMV-encoded m41.1 targets BAK function as the viral inhibitor of BAK oligomerization (vIBO) [172]. Comparable activities are conserved in homologous genes of GPCMV [174,175] and, based on sequence similarity, across known rodent betaherpesviruses [169]. When disrupted individually, m38.5 or m41.1 single mutant viruses do not show significant defects in replication in cultured cells and only a modest fitness compromise in cultured macrophages or during infection of mice [176,177,178]. Double mutants disrupting both m38.5 and m41.1 function are also replication competent in most cultured cells but show dramatic attenuation in macrophages and mice, supporting a crucial role for sequestering BAX and BAK function in viral pathogenesis [134]. Studies with these MCMV orthologs also implicate serine protease HtrA2/Omi cell death analogous to vMIA suppression of this pathway in HCMV-infected cells. HtrA2/Omi germ line gene disruption in mice results in a striking neurodegenerative compromise [168,179] that is reversed by restoring brain-specific expression of HtrA2/Omi [180]. Such rescued mutant mice are fertile and viable but experience premature aging. While the full complexity of cell death suppressors and the death pathways they inhibit has not been fully explored with either HCMV or MCMV, studies with MCMV m38.5/m41.1 mutants reinforce the biological conservation of cell death pathways and virus-encoded cell death suppressors that have been adapted during evolution [134]. It is particularly striking that mitochondrial serine protease HtrA2/Omi-dependent death plays out with HCMV UL37x1 single mutant or MCMV m38.5/m41.1 double mutant viruses [134,154,157] but has otherwise been overlooked as an independent programmed death pathway [111]. Given common properties of MCMV and HCMV, the HtrA2/Omi-dependent cmvPCD pathway is evolutionarily ancient, possibly originating in the survival of eubacteria, and certainly conserved over at least 80 million years of mammalian evolution.

A long noncoding (lnc)RNA referred to as RNA2.7 or β2.7 is produced by HCMV from very early times of infection [27,181]. This unspliced transcript [181,182,183] retains translation repression signals [184] but can be adapted to support high-level expression of foreign proteins from recombinant HCMV [185]. Surprisingly, functionally similar lncRNA is not found in other CMVs or herpesviruses. Even though disruption of RNA2.7 expression does not impact levels of viral replication in fibroblasts [186], the RNA interacts with mitochondrial respiratory complex I to counteract oxidative stress by maintaining membrane potential [187]. Complex I plays a central role in metabolic energy production [188] and response to stress [189]. RNA2.7 efficiently stabilizes complex I in a variety of cell types [187,190,191] and may support latent infection [192,193]. By deploying RNA2.7, HCMV targets mitochondrial function through a BCL2 family member-independent mechanism.

## 5. Inhibitors of Extrinsic Apoptosis

TNF family death receptor signaling has long been implicated in antiviral host defense as well as CMV pathogenesis [194,195,196]. The HCMV UL36-encoded vICA, an immediate early protein [135], targets this key mediator of extrinsic apoptosis, binding directly to the death domain of procaspase-8 to prevent autoactivation [131,146]. In this manner, vICA suppresses any contribution of caspase-8 to TNF family death receptor signaling [147,197]. From what is known caspase-8 activation by death receptor-independent pathways [64,198] should also be fully prevented. UL36 has been highly conserved during evolution with sequence homologs in all betaherpesviruses. Thus, HCMV UL36 and MCMV M36 function similarly as inhibitors of caspase-8 that function to prevent cell death, dictate host immune response patterns, and contribute to the resistance of infected cells to cytotoxic T cell killing [104,146,199,200,201,202]. In addition, HCMV vICA contributes to resistance to caspase-independent death during infection [199] being implicated in the suppression of virus-induced RIPK3-dependent necroptosis [59,203]. MCMV M36 does not carry out these additional activities [204] and predictably, sensitizes cells to RIPK3-dependent necroptosis by blocking caspase-8 activity [103]. In either MCMV or HCMV, vICA appears critical to infection of myeloid cells [146,199], and studies with MCMV M36 mutant viruses implicate control over TNF-dependent cell death in a variety of cell types [202]. Myeloid cells produce TNF that triggers apoptosis in either myeloid (autocrine) or non-myeloid cells (paracrine) during infection, and this outcome is completely blocked by vICA-expressing MCMV. M36 vICA-mediated caspase-8 suppression unleashes necroptosis during infection [103,104]; whereas, HCMV vICA does not [203]. This difference is ascribed to a striking species-specific ability of the latter to interact and interfere with MLKL to prevent the execution of necroptosis [203,204]. HCMV also regulates the host-encoded caspase-8 suppressor, cellular FLICE-like inhibitory protein (cFLIP) in studies focused on human retinal pigment epithelial cells [205,206]. While evidence suggests HCMV-encoded immediate early 2 protein regulates cFLIP, interpretation is again confounded by the absence of functional vICA in the viral strain chosen to study. Overall, vICA plays a key role in CMV-mediated subversion of extrinsic apoptosis, with additional impacts on caspase-independent and cell death-independent consequences of caspase-8 signal transduction.

CMVs also subvert cell surface expression of certain TNF family death receptors. HCMV UL141-encoded protein binds to TNF-associated ligand-inducing apoptosis (TRAIL) death receptors to restrict signal transduction and natural killer (NK) cell–host defense [207]. The importance of TRAIL signaling suppression in CMV pathogenesis was revealed in studies of MCMV-encoded m166, which inhibits TRAIL receptor expression [208]. Similarly to other TNF family death receptors, TRAIL receptor signal transduction includes both death-dependent and death-independent outcomes [209]. Mutant viruses lacking m166 exhibit severely compromised replication in mice, most notably in the liver, which is restored by depleting NK cells [208]. Further infection of TRAIL receptor-deficient mice restores m166 mutant virus replication, thereby providing strong genetic evidence supporting TRAIL signal transduction in control of antiviral host defense.

## 6. Inhibitors of Lytic Cell Death Pathways, Necroptosis and Pyroptosis

Pathogen sensing leads to proinflammatory cell death outcomes that contribute to host defense as well as disease pathogenesis due to the proinflammatory consequences of releasing cell components within tissues [113,116]. Two recognized programmed cell death pathways result in a loss of plasma membrane integrity due to pore formation, cell swelling, and rupture. Pyroptosis is executed by gasdermins [115] and necroptosis is executed by MLKL [210]. These pathways proceed independently of apoptotic signal transduction without any contributions from apoptotic caspases (executioner caspase-3, -6, -7 or autoactivating caspase-8 and -9). Necroptosis is unleashed as an alternative to extrinsic apoptosis when caspase-8 activity is compromised, a common occurrence during infection with DNA viruses that deploy specific cell death suppressors such as CMV vICA [19]. Inflammasome signaling and pyroptosis are important in bacterial pathogenesis [112,113,114] although the double-stranded DNA (dsDNA) sensor absent in melanoma 2 (AIM2) senses MCMV infection (as well as other viral and intracellular bacterial pathogens) to drive inflammasome activation and pyroptosis [211]. AIM2 function is countered by MCMV-encoded M84, a recently characterized suppressor of inflammasome activation and caspase-1-mediated pyroptosis [110]. M84 interacts with pyrin domain-containing adaptors AIM2, ASC, IFI203, and IFI204. M84-deficient MCMV replication proceeds poorly in macrophages dependent on the presence of AIM2, ASC, and caspase-1, due to increased inflammatory cytokine production and pyroptosis, indicating that the AIM2 inflammasome becomes more active in the absence of this viral modulator. While M84 has amino acid sequence similarity to two HCMV-encoded genes (UL83 and UL84) and, although HCMV-encoded UL83 protein has been reported to impact sensing and signaling of AIM2 as well as DNA sensor IFI16 [212,213], additional studies are needed to determine whether these genes act in a parallel manner. Induction and suppression of virus-induced necroptosis and pyroptosis during infection with CMVs have been recently reviewed [68,70].

Necroptosis is a regulated cell death pathway triggered by the recruitment of RIPK3 by RIPK1, initially characterized downstream of TNF death receptor signaling carried out in the presence of caspase-8 inhibitors [214,215,216]. RIPK1-dependent recruitment of RIPK3 depends on RIP homotypic interacting motif (RHIM) interactions [217] regulated by posttranslational modification and cut short by caspase-8-mediated cleavage of RIPK1 and possibly RIPK3 [218,219]. Two additional RHIM-containing signaling adaptors recruit RIPK3 and trigger necroptosis, TIR domain-containing adaptor molecule 1 (TICAM1, commonly called TRIF), best known as an adapter in TLR3 and TLR4 signaling [220,221] and Z-nucleic acid (Z-NA) binding protein (ZBP)1 (also called DLM1 or DAI) [222,223,224,225], whose RHIM was discovered in the Mocarski laboratory [224] following the identification of ZBP1 as a pathogen sensor [225], and whose role in necroptosis emerged through the study of MCMV M45 RHIM function [226,227,228]. M45 is an enzymatically inactive homolog of a large subunit of ribonucleotide reductase (R1) with an amino-terminal RHIM that blocks activation of RIPK3 as a viral inhibitor of RIP activation (vIRA) [221,226,227,228,229]. The initial interpretation that M45 controlled MCMV tropism for endothelial cells [230] became clarified when the existence of the RHIM and the biological importance of inhibition was unveiled [226,227]. M45 RHIM-deficient MCMV fails to replicate in mice due to the induction of ZBP1-RIPK3-MLKL necroptosis [227,228]. These studies highlight the exceptional potency of necroptosis as an innate host defense pathway that can prevent infection of the natural host animal. RIPK3 in cells dictates susceptibility to necroptosis and is rapidly lost when mouse and, particularly, human cells are placed in culture or when cancer cell lines are employed [215]. When RIPK3 levels are too low or absent, phosphorylation of executioner MLKL fails to take place [231,232]. Virus-induced necroptosis has been recognized in other herpesviruses and poxviruses that encode inhibitors [233,234] as well as in influenza viruses which do not [235,236]. This lytic cell death pathway was slow to be identified due to the common loss of ZBP1, RIPK3, and MLKL in cultured cells and the absence in different chordate species [237] as well as the variable requirements such as interferon stimulation to drive ZBP1 expression [238]. In all studied viral systems newly synthesized Z-RNA acts as the PAMP engaging ZBP1 to initiate cell death pathways [68,239]. RIPK3 has the potential to drive extrinsic apoptosis [101,102,240] as well as inflammasome-mediated inflammatory pathways and allied caspase-1-dependent pyroptosis [64,241,242,243,244,245]. The potential for restricted caspase-8 activity to unleash necroptosis depends on many factors, including cell type. Suppression of caspase-8 by MCMV vICA sensitizes to ZBP1-dependent, virus-induced necroptosis [104,246]. MCMV-infected macrophages also engage a slower form of necrotic death independent of pathogen sensor ZBP1 [104]. The specific cell death signaling pathway triggered likely varies depending on the balance of signaling components. Studies of CMV-encoded, herpes simplex virus (HSV)-encoded, and poxvirus-encoded cell death suppressors support the view that cell death pathways evolved to become more elaborate and interdependent in response to virus-encoded cell death suppressors [68,72]. The constellation of cell death suppressors deployed by pathogens provides insights into the evolution of cell death pathways in support of host defense [68].

MCMV M45 mutant virus-induced necroptosis proceeds independently of both RIPK1 and TRIF [227,228]. Although the HCMV-encoded R1 homolog, UL45 lacks an amino-terminal RHIM and cannot block necroptosis [59], HCMV UL36-encoded vICA inhibits necroptosis by interacting with executioner MLKL in addition to inhibiting caspase-8 apoptosis [203]. M45-mediated modulation of RIPK1 also impacts NF-κB-dependent cytokine activation during infection with MCMV [247,248,249] as well as with HCMV [250,251]. Thus, M45 makes an important secondary contribution to control over NF-κB function [252,253].

During MCMV infection, ZBP1 recognizes newly synthesized viral RNA [254,255], a pathogen recognition step that also occurs with other herpesviruses [233,256], poxviruses [238,257] and influenza viruses [235,236,258]. Notably, influenza virus infection reportedly drives multiple death pathways (combined apoptosis and necroptosis or PANoptosis), but this outcome is not observed with mutant herpesviruses lacking inhibitors of caspase-8 and RHIM signaling [104,233]. During infection with the vaccinia virus, ZBP1 is activated by Z-form RNA, dependent on the concerted function of two distinct dsRNA binding domains [238]. Like dsDNA, dsRNA physiologically takes on a right-handed double-stranded helix, called A-RNA, but can be induced to form a left-handed conformation by binding to a Zα motif within a Z-NA binding domain found in ZBP1 as well as vaccinia E3 [239]. Vaccinia E3 protein consists of an amino-terminal Zα-containing Z-NA binding domain as well as a carboxyl-terminal dsRNA binding domain. Z-RNA formation during viral infection is promoted by the carboxyl-terminal dsRNA binding domain acting in concert with the amino-terminal Z-NA binding domain to prevent recruitment of Z-RNA by ZBP1 and activation of necroptosis [238,239]. In MCMV, vIRA prevents recruitment of RIPK3 by ZBP1, directly competing with this RIPK homology interaction motif (RHIM)-dependent step [59,224,228,229]. In influenza, defective viral genomes bound by nucleocapsid protein are sensed by ZBP1 [236]. Z-RNA formation during influenza, MCMV, or HSV infection are likely to involve additional viral dsRNA binding proteins. The parallels between rodent betaherpesviruses and primate alphaherpesviruses are striking due to a conserved amino-terminal RHIM within their R1 proteins. The R1 (ICP6) encoded by herpes simplex virus (HSV)-1 and HSV-2 blocks necroptosis in human cells [234,259] and naturally prevents virus-induced necroptosis triggered very early after infection of human cells by preventing ZBP1 recruitment of RIPK3 using a mechanism very much like MCMV M45 [233]. HSV ICP6 directly triggers necroptosis by directly recruiting RIPK3 in mouse cells and mice [259,260,261], a species-specific RHIM-dependent pathway that may be blocked by substituting the M45 RHIM for the ICP6 RHIM in HSV-1 [259]. This work has been extensively reviewed [67,68,72,246,262,263] and may depend on differences in the requirement for RIPK1 for ZBP1-RIPK3 signaling in human versus mouse cells [264]. Mechanistic differences that control positive and negative RHIM signaling outcomes remain unresolved.

## 7. Conclusions and Perspective

Programmed cell death contributes to host control at the earliest stages of virus infection. Virus-induced apoptosis, necroptosis, and pyroptosis may be triggered via host sensors of viral nucleic acids as well as through the stress of infection. A temporal and cell-type hierarchy of alternative cell death pathways emerges from observations in herpesviruses and poxviruses that express pathway-specific cell death suppressors, whereas the notion of combined pathways has emerged from studies with influenza viruses. Timing is crucial. Death of infected cells before the production of progeny virus starts clearly benefits the host. The more delayed the death pathway, the better it is for virus pathogenesis. Continued studies of the host defense potential of cell death pathways in model mammals as well as humans should resolve outstanding issues and provide insights into this ancient type of cell-intrinsic immune mechanism.
